# Rural libraries implementing walking groups or walking groups plus civic engagement for walkability in rural communities: a comparative effectiveness trial study protocol

**DOI:** 10.1186/s12889-023-16788-0

**Published:** 2023-10-02

**Authors:** Cynthia K Perry, Rebecca Seguin-Fowler, Jay E. Maddock, Noah Lenstra, Nathan F. Dieckmann, Jessica Currier, Elena Andreyeva, Jim Winkle, Stewart G. Trost

**Affiliations:** 1 Oregon Health & Science University School of Nursing, 3455 SW US Veterans Hospital Rd, Portland, OR 97239 USA; 2 Texas A & M University Institute for Advancing Health through Agriculture, 1500 Research Parkway, Centeq Building B, College Station, TX 77845 USA; 3 Texas A & M University School of Public Health, 1266 TAMU, College Station, TX 77843 USA; 4 University North Carolina Greensboro School of Education, 1300 Spring Garden St, Greensboro, NC 27412 USA; 5 Knight Cancer Institute, Division of Oncological Sciences, Oregon Health & Science University, 2720 S. Moody Ave, Portland, OR 97201 USA; 6 Texas A & M University, 212 Adriance Lab Rd, College Station, TX 77843 USA; 7 School of Human Movement and Nutrition Sciences, The University of Queensland, Brisbane, Qld 4072 Australia

**Keywords:** Walking, Rural, Comparative effectiveness trial, Physical activity, Civic engagement

## Abstract

**Background:**

Rural residents generally lack adequate physical activity to benefit health and reduce disparities in chronic diseases, such as cardiovascular disease and certain cancers. The Socioecological Model describes physical activity as involving a dynamic and reciprocal interaction between individual, social, and community factors. Community group-based walking programs and civic engagement interventions aimed at enhancing physical activity have been successful in rural communities but have not targeted all three socioecological levels. Public libraries can act as innovative public health partners in rural communities. However, challenges remain because rural libraries often lack the capacity to implement evidence-based health promotion programming. The goals of this study are (1) build the capacity for rural libraries to implement evidence-based health promotion programs, (2) compare changes in physical activity between a group-based walking program and a combined group-based walking and civic engagement program with rural residents, and (3) conduct an implementation evaluation.

**Methods:**

We will conduct a comparative effectiveness study of a group-based walking (standard approach) versus a group-based walking plus civic engagement program (combined approach) aimed at enhancing walkability to increase physical activity among rural adults. Key mediators between the program effects and change in outcomes will also be identified. Finally, we will evaluate program implementation, conduct a cost effectiveness evaluation, and use a positive deviance analysis to understand experiences of high and low changers on key outcomes. Twenty towns will be matched and randomized to one of the two conditions and our aim is to enroll a total of 350–400 rural residents (15–20 per town). Study outcomes will be assessed at baseline, and 6, 12, and 24 months.

**Discussion:**

This study will build the capacity of rural libraries to implement evidence-based walking programs as well as other health promotion programs in their communities. The study results will answer questions regarding the relative effectiveness and cost effectiveness of two multilevel physical activity interventions targeting rural communities. We will learn what works and how these multilevel interventions can be implemented in rural populations.

**Trial Registration:**

ClinicalTrials.gov Identifier: NCT05677906.

**Supplementary Information:**

The online version contains supplementary material available at 10.1186/s12889-023-16788-0.

## Background

Engaging in the recommended amount of physical activity (150 min per week of moderate level activity) has numerous health benefits, including lower risk of chronic diseases, such as heart disease and diabetes and some types of cancer [1]. Worldwide, achieving enough physical activity could prevent upwards of 5.3 million deaths per year [2]. Despite this evidence, nearly 75% of US adults fail to achieve adequate levels of physical activity [3]. Rural residents are less likely to meet recommended levels of physical activity compared with their urban counterparts [4] and tend to have higher rates of obesity, cancer and heart disease compared with their urban counterparts [5–7]. Walking is the most common form of physical activity [8–10], and people who walk are more likely to meet recommended levels of physical activity compared to non-walkers [8]. However, rural residents compared with urban residents report walking less [11]. The persistence of this rural-urban disparity in physical activity suggests a need for effective interventions for rural communities.

The Socioecological Model (SEM) is a framework for understanding determinants of health behaviors including physical activity. The model emphasizes the reciprocal interplay among multiple levels of influence: individual, social, and community [12]. The SEM is consistent with social cognitive theory (SCT), which posits that health behavior is based on the dynamic and reciprocal interaction between individual (e.g., self-efficacy, self-monitoring), social (e.g., modeling, support, cohesion), and environmental (e.g., built environment) factors. Interventions designed to promote physical activity are likely to be most effective if they target individual, social, and community factors [12]. In 2015, the US Surgeon General issued a *Call to Action* to promote walking and walkable communities highlighting the importance of walking and having safe places to walk [13]. Recognizing the impact of the environment on health, the Centers for Disease Control and Prevention recommend changing the social and built environments to make the healthy choice easier [14].

Community group-based walking programs have been shown to increase walking, physical activity, and fitness and improve health outcomes, including with rural communities [15–18]. Walking groups that last longer than six months have been found to have larger effect sizes, suggesting that benefits appear to be durable over time [19, 20]. Group-based walking programs address social influences of physical activity by building group cohesion, social support, and social capital to promote adoption and maintenance of walking for health [18, 21, 22]. In rural communities, social support (or lack of social support) can be an important facilitator (or barrier) for walking [23–25], and group-based walking programs can strengthen social networks and improve social support [26, 27]. The built environment (e.g., sidewalks, traffic) is also associated with walking [28, 29]. Walking groups can impact perceptions of the built environment [30] and aspects of the built environment can influence adherence to walking program [31, 32].

Civic engagement is one approach to built environment change, such as enhancing walkability. Civic engagement refers to individuals participating in the community in order to improve the conditions of the community [33]. Civic engagement for built environment change focuses on the social and community factors and can improve local built environment and policies related to community physical activity [34–36]. At the interpersonal level, civic engagement builds social support and collective efficacy to accomplish community change [37].

Relative to urban communities, rural communities generally lack the necessary resources to implement public health programming. An innovative public health partner in rural communities is public libraries, which are increasingly offering a multitude of programs [38–43]. A 2017 survey found that 42% of libraries surveyed offered general walking, hiking, bicycling, or running programs [40]. Libraries are also implementing initiatives to engage community members in civic engagement [44]. Although libraries may be ideal institutes to lead civic engagement efforts, no published studies of civic engagement for walkability have been based in libraries since libraries often lack the capacity to implement evidence-based programming. Capacity building is one strategy that can address this gap and provide support to libraries for implementation and evaluation so that programs reach the intended population and achieve the desired results [45].

Although rural residents face greater health disparities compared to urban residents in terms of chronic disease, there is a major gap in achieving reach and sustainable impact in behavior changes to reduce the risk of these diseases. Multilevel physical activity interventions are likely to be more effective, but there is limited evidence on how they work with rural communities, which often lack the capacity to conduct and sustain these interventions. Thus, disparities in rural physical activity persist. This study addresses this gap by comparing the effectiveness of multilevel physical activity programs that promote walking in rural communities and identifying key mediators of the program. The aims of this study are (1) increase the capacity of rural libraires to deliver evidence-based health promotion programs, (2) compare the effects of a group-based walking program with a combined group-based plus civic engagement program on physical activity, cardiovascular fitness and collective efficacy among rural residents, 2a) identify key mediators between the intervention program effects and change in physical activity, fitness, and collective efficacy, and (3) evaluate program implementation and cost effectiveness.

## Methods

### Design and setting

This study is a comparative effectiveness experiment of a group-based walking program, Step It Up!, versus a combined program of both Step It Up! (SIU) and a civic engagement program aimed at walkability, the Change Club. We also will conduct a mixed-method implementation evaluation. At the organizational level (library), a prospective convergent parallel mixed methods design [46] will be used, in which quantitative and qualitative data will be collected in parallel, analyzed separately, and merged in order to gain a full understanding of factors that influence implementation and sustainability of the program in the libraries. At the participant level, a positive deviance approach will be used to understand experiences of high and low changers on outcomes. A sequential mixed methods design [46] will be used, with quantitative participant outcomes informing selection of participants for qualitative work.

This study will take place in 20 rural communities in Oregon. The US Institute of Museum & Library Services (IMLS) classifies libraries as serving urban, suburban, town, or rural areas using the National Center for Education Statistics (NCES) definitions for each classification [47]. The library’s classification is based on the geocoded latitude and longitude of the library’s address. For this study, libraries serving towns and rural areas are eligible. The NCES, drawing upon the U.S. Census, defines towns as areas outside of urbanized areas, but inside towns with populations between 2,500 and 50,000. This grouping of rural and town libraries follows the standard practices of libraries, as seen in organizations like the Association for Rural & Small Libraries.

The rural communities in which the libraries are located will be matched on population, median household income, percent of residents who identify as white, walk score, rate of violent crime, percent of population 65 years or older, employment rate, percent residents without health insurance, and area deprivation index. Matched pairs will then be randomized to either a group-based walking program, Step It Up! (SIU) or the combined program (Combined) that includes both SIU and a civic engagement program, Change Club. Cluster (pairs) randomization will be implemented using the Goldilocks approach [48]. This approach involves iteratively choosing pairs that minimize distance with respect to potential confounding variables, visually assessing differences across possible randomizations, and reweighting variables by selecting confounder weights. The best pair matching was achieved by unit weighting (given weight = 1) all confounders except for population (weight = 3), walk score (weight = 3), area deprivation index (weight = 3), and percent white only (weight = 2). Libraries will be randomized prior to the start of the capacity building and training so that librarians in the SIU group are not exposed to the Change Club curriculum.

We will disseminate results to the scientific community through peer reviewed publications and presentations and to the communities through presentations and written materials, such as an infographic.

### Study aims and approaches

#### Aim 1

Increase the capacity of rural libraries to deliver evidence-based health promotion programs

Capacity building will start pre-program implementation to get the libraries ready for implementation and will continue throughout implementation. We will provide the 20 librarians with technical assistance on program implementation and $5,000 stipends to support program implementation. Librarians will be divided in two groups based on the randomization, a SIU group and a Combined group and will attend trainings and webinars with their group. We will hold two 60-minute trainings for the SIU program and an additional 60-minute training for the Combined program prior to program implementation. All librarians will receive a SIU Toolkit that provides information on how to set up and deliver a group-based walking program as well as handouts for participants. Librarians in the Combined Program will be given a facilitator guide and curriculum for the Change Club and handouts for participants. Additionally, the Change Club groups will be awarded $5,000 after submitting a budget for use of the funds. We will hold monthly webinars to provide an opportunity for the librarians to learn from and support each other as they plan for and implement the programs. The research team will be available via phone calls or email to librarians throughout the study for questions or individualized support as needed. Each librarian will complete the Public Health Professional Competency Assessment [49] prior to the trainings and at the end of the two-year study.

#### Aim 2

Compare the effects of a group-based walking program with a combined group-based plus civic engagement program on physical activity, cardiovascular fitness, and collective efficacy among rural residents.

#### Sub-aim 2a

Identify key mediators between the intervention program effects and change in physical activity, fitness, and collective efficacy.

The objective for Aim 2 is to determine the amplified effects of the combined program on outcomes. Our working hypothesis is that participants in the Combined program will have greater improvement in outcomes compared with SIU program alone because participants in the Combined Program will engage in activities directed at individual, social, and community factors.

### Participants

Librarians, with assistance from the research team, will recruit 15–20 participants. Inclusion criteria are age 18 or older, inactive (defined as engaging in physical activity < 3 days per week), ability to walk for at least 20 min, living within the rural community served by the local library, and ability to travel to the local library/location of walking group. Each potential participant will complete the Physical Activity Readiness-Questionnaire (PAR-Q) [50] as part of the screening process to ensure that they are able to participate in walking groups. If the potential participant answers “yes” to any PAR-Q question, they will be asked to obtain healthcare provider authorization to participate. Based on our prior experience in similar populations and settings, we anticipate that this will be non-limiting to recruitment, while ensuring human subject protection. Exclusion criteria include participation/intention to participate in other lifestyle modification program(s), cognitive impairment, inability to communicate due to severe uncorrected hearing loss or speech disorder, and/or severe visual impairment (if precludes completion of assessments and/or intervention).

Recruitment methods will include fliers/postcards/business cards and bookmarks placed at community locations, such as grocery stores, banks, and retail establishments; word of mouth; radio and/or newspaper ads; and press releases sent to local newspapers. Fliers/postcards/business cards or information as worded on the fliers /post cards/business cards may also be posted on the library or city/town government websites and included in printed and electronic versions of library or community organization newsletters.

### Interventions

The Socioecological Model and Social Cognitive Theory serve as the theoretical foundation for both SIU and the Change Club. We hypothesize that program effects are mediated by increased self-efficacy, social support, group cohesion, and perceived environment resulting in increased physical activity, fitness, and collective efficacy (belief in group capability to achieve goal) [51]. Rural librarians will lead the group walk for SIU, and the group walk and Change Club session for the Combined program. After six months, group walks and Change Club sessions will be peer-led to enhance sustainability. The programs will run for two years.

### Step it up!

Group walks will occur weekly for 60 min. Participants will progressively build up to walking for 45 min at a brisk pace. The first walk will start with setting group norms, building rapport, and developing Specific Measurable Attainable Relevant Timely (SMART) physical activity goals and a short walk. For the next four weeks, the walk will start with a check-in, followed by a 30-minute walk and end with brief stretching. Thereafter, the walks will start with a check-in followed by a 45-minute walk and end with brief stretching. The librarian will use behavior change strategies (Table 1) and will use the spirit of motivational interviewing when talking with participants about behavior change. Each group will create a group name and be given group t-shirts. Each participant will be given a Fitbit Inspire for monitoring and motivation.

**Table 1:** Step it up! socioecological & behavior change theory-based strategies

Strategies	Underlying Theoretical Concepts	Empirical Rationale Impact on Study Outcomes
***Individual Domain***		
Discuss feelings regarding exercise experiences	• Commitment • Supportive relationships • Emotional & informational support	Increases group cohesion, social support, exercise adherence [52–55]
Discuss substituting walking for sedentary behavior	• Emotional & informational support	Increases self-efficacy, social support, adherence [52, 56]
Establish goals	• Self-control	Increases self-efficacy, exercise adherence [52, 56]
Fitbit log	• Self-monitoring • Self-reinforcement	Increases self-efficacy, exercise adherence [52, 56]
Emphasize positive benefits	• Physiologic responses • Consciousness raising • Intrinsic motivation	Increases self-efficacy, exercise adherence [52, 54, 57]
Participatory	• Autonomy	Increases self-efficacy, exercise adherence [52, 54, 56, 57]
***Social Domain***		
Positive feedback from group leader	• Positive reinforcement	Increases social support, self-efficacy, group cohesion, exercise adherence [52–54, 57, 58]
Positive feedback from group members	• Social persuasion • Emotional & informational support	Increases self-efficacy, group cohesion, exercise adherence [52, 53, 55, 58, 59]
Exercise together as group	• Modeling experiences • Mastery experiences	Increases self-efficacy, collective efficacy, exercise adherence [52]
Group t-shirt	• Group identity	Increases group cohesion & connectedness [52, 53, 59]

### Combined program

The SIU group walk will be implemented as described above. The group will meet for the Change Club for 30 min after the group walk. The Change Club curriculum has been revised to be implemented in 30-minute sessions and to focus on walkability. The first month topics include building group rapport and identity and establishing group norms, as well as assessment of community walkability assets (Table 2). Months 2–3 focus on change objective identification (e.g., improve trail access), identification of potential partners, and action planning, including delineating benchmarks. The remaining three months, the Change Club will execute the action plan to implement the change, overcoming barriers and challenges. Each Change Club will receive $5,000 upon submitting a proposed budget.

**Table 2:** Socioecological aspects of change club curriculum

	*Social Domain*	*Community Domain*
Month 1: Unity & Togetherness	Building rapport; establishing purpose; deciding group name & expectations	Community walking tour; discussing assets, barriers, resources
Month 2: Community Needs	Gaining social support strategies; defining purpose & goals for group	Interviewing local leaders; identifying town needs
Month 3: Next Steps	Leadership skills; identifying connections & strengths of members	Developing action plans; delegating tasks & action items for group members
Ongoing	Overcoming sabotaging related to physical activity	Executing action plan; reviewing progress, benchmarks, accomplishments

### Data Collection

**Fig. 1:**
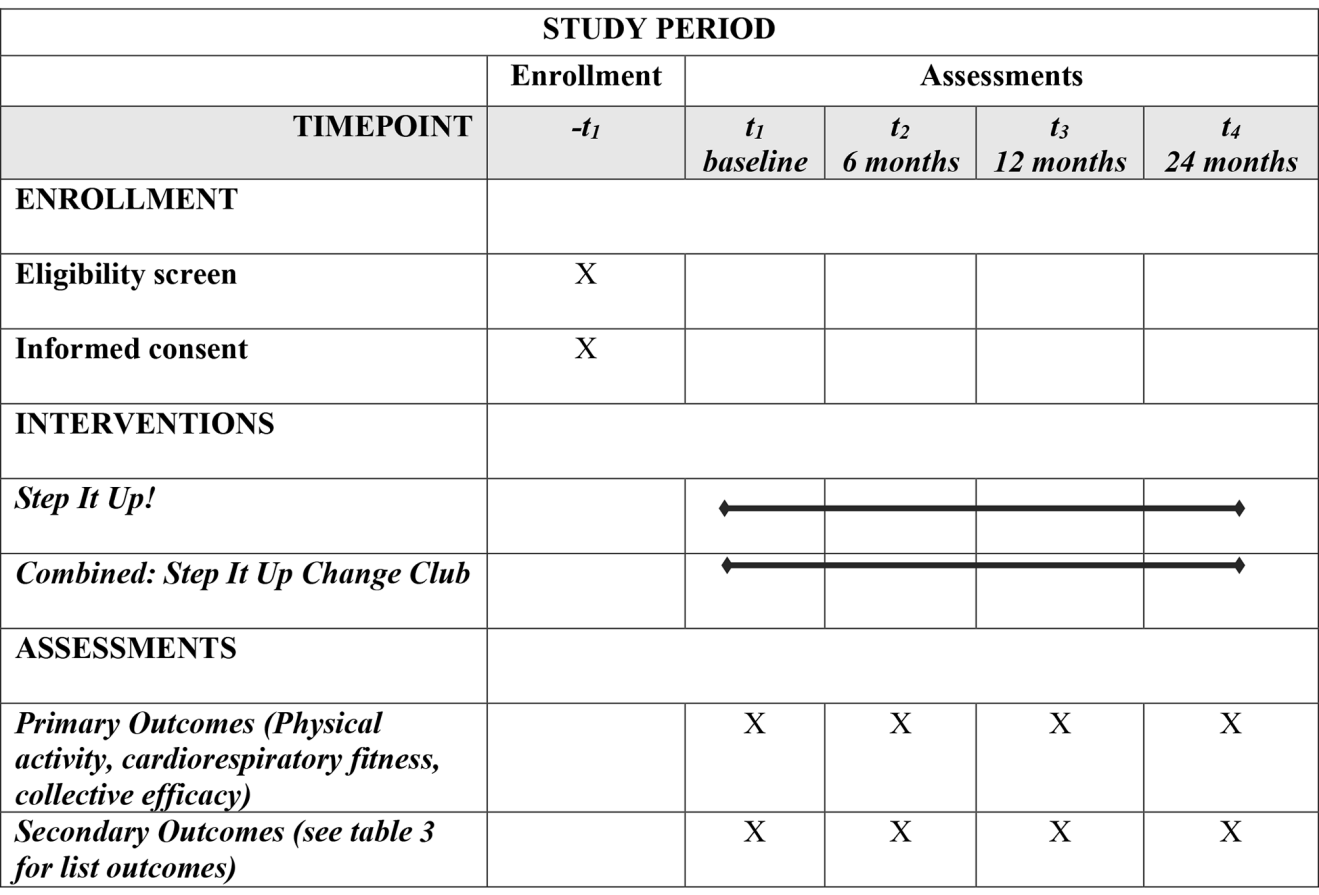
Schedule intervention and assessments

Data will be collected at four timepoints (baseline and 6, 12, and 24 months) by trained research staff. Data collection events will be scheduled at each library at each of the timepoints. The SPIRIT flow diagram (Fig. 1) provides the timing of the intervention and assessments for the trial.

### Primary outcomes

#### Physical activity

Physical activity will be assessed with an ActiGraph GT3X + accelerometer (ActiGraph Corporation, Pensacola, FL, USA), worn on the non-dominant wrist for seven consecutive days. Raw accelerometer data collected at 30 Hz will be processed into physical activity metrics using a machine-learned random forest classifier specifically designed and validated for assessing movement behaviors in free living adults [59]. The random forest classification model uses features in the raw acceleration signal to classify each 10 s window (epoch) as sedentary (lying or sitting still), stationary plus (active sitting, standing still, active standing), walking, or running. Predictions for each 10 s period will be smoothed using a modal filter spanning two lead and two lag epochs. In the current study, time spend in moderate-to-vigorous intensity PA will be defined as the sum of daily time spent walking and running. Non-wear periods will be identified by summing the 10-sec windows in which the standard deviation of the acceleration signal vector magnitude is < 13 mg for > = 30 consecutive minutes [60]. The participants accelerometer data will be included in the analyses if they have ≥ 5 days (with at least 1 weekend day) in which wear time is 10 h or longer [61].

#### Cardiorespiratory fitness

The six-minute walk test will measure cardiorespiratory fitness [62]. Participants will walk as quickly as they can on a marked route for six minutes. Distance will be measured in meters.

#### Collective efficacy

The Collective Efficacy Scale will be used to measure collective efficacy [63]. This survey has two subscales- informal social control which has four questions and statements that respondents rate on a 5-point Likert scale from very 1 = likely to 5 = very unlikely and social cohesion and trust, which has four statements that respondents rate on a 5-point Likert scale from 1 = strongly agree to 5 = strongly disagree.

### Secondary outcomes

We will measure height, weight, waist and hip circumference, blood pressure, and resting pulse. Each measurement will be taken twice. Participants will complete surveys that include individual, social, and community level questionaries (Table 3).

**Table 3:** Secondary outcomes, self-report surveys

Outcome	Measure	Number of Questions	
***Individual Level***			
Demographics	Demographics (e.g., age, gender, race/ethnicity)	9	
General Health	SF-36 [65] and BRFSS [66]	13	
Physical Activity	International Physical Activity Questionnaire [67]	27	
Physical Activity Self-Efficacy	Self-efficacy Exercise Behaviors Scale [68]	5	
***Social Level***			
Group Cohesion and Connectedness	Physical Activity Group Environment Questionnaire [69]	17	
Social Support	Social Support for Exercise Scale [70]	13	
Collective Efficacy	Collective Efficacy Scale [64]	8	
Mobilization	Mobilization Scale Individual [71]	8	
***Community Level***			
Civic Engagement Attitudes	Civic Engagement Scale [72]	14	
Social Norms	Social Norms for Physical Activity [73]	5	
Walking Environment	Neighborhood Environment Scale [74]	8	
Greenspace Exposure	The People and Nature Survey [75]	3	

BRFSS: Behavioral Risk Factor Surveillance Survey. SF-36: Short Form 36

### Sample size calculation

Results of multiple studies have shown that walking group interventions have small to large effects on physical activity. What is unclear is how much advantage can be gained by combining the walking group and civic engagement interventions. To be conservative, we chose to power this study to detect small effect sizes (Cohen’s d ~ 0.20). We calculated the minimum detectable effect (MDE) for the primary cross-level interaction effect testing whether outcome changes across time differs between intervention groups. MDE was estimated using a multi-level framework using Optimal Design software [75] for the planned intention-to-treat (ITT) analyses for the primary outcomes. Multi-level models were used for power analyses using a range of ICCs and all power analyses assumed alpha = 0.05, power = 0.80, continuous normal outcomes, complete cases, two intervention groups, and four time points (baseline-24 months). We estimate that approximately N = 150 participants per intervention group will allow us to detect effect sizes in the small range (Cohen’s d = 0.15-0.25). Given an expected attrition rate of approximately 10% per year, we will recruit 175–200 participants per arm.

### Data management and analysis

Survey data will be collected via REDCap, a highly secure and robust web-based research data collection and management system [76]. Anthropometric measurements and 6-minute walk data will be recorded in Excel and accelerometer data will be downloaded into actilife software; these data files will be stored on a secure cloud-based storage system requiring two-factor authentication.

Standard descriptive statistics of frequency, central tendency, and dispersion will be used to describe the sample and standard psychometric analyses (e.g., Cronbach’s alpha) will be used to assess the reliability of all self-report measures. Before formal modeling, we will identify any departures from statistical assumptions (e.g., outliers, skewness). All primary analyses for each aim will be conducted ITT (including all participants who were randomly assigned to condition regardless of percentage of intervention completed). In addition, we will explore adherence-based analyses in which we will compare groups including only participants who completed 70% or more of the intervention after controlling for any covariates related to adherence.

Our primary aim will be tested with a linear mixed-effects (multi-level) modeling approach implemented in the lmer package in R. Within this framework, the level-1 and level-2 models will include within-person (e.g., measures across time) and between-person (e.g., intervention assignment) variables, respectively. An additional level will be added to the model to account for nesting within town. Changes in outcomes across time will be modeled as a fixed time effect and model testing will be conducted to determine how best to represent change in each outcome (e.g., linear versus nonlinear). Intervention effects will be examined by specifying fixed-effect dummy variables that compare the intervention groups. Specific hypothesis testing will be by specifying cross-level interactions (time x group contrasts) to determine whether differences across time (e.g., baseline-24 months) significantly vary between intervention groups. Model testing will be used to determine the appropriate random effects structure for each outcome, with the default model including random slopes and intercepts. We will evaluate primary hypothesis tests at traditional significance levels (alpha = 0.05) and use modern methods to control for false discovery rate [77] for post hoc analyses. We will focus interpretation on effect size estimation with accompanying 95% confidence intervals (e.g., Cohen’s d). Model diagnostics (e.g., examining residual/predicted value plots) will be used to confirm statistical assumptions have been met. In the case of violations, more robust alternative models will be employed (e.g., robust mixed-effects models) as appropriate.

We will examine dropout and patterns of missing data to determine mechanisms (missing completely at random [MCAR], missing at random [MAR], or not ignorable) [78]. In the case of outcome data MCAR or MAR, model-based maximum likelihood estimation underlying the mixed-effects modeling approach (described above) will allow unbiased parameter estimation using all available data (i.e., missing data are handled efficiently with no loss of information). In the case of predictor/covariate data MAR, we will employ principled methods of multiple imputation available in several R packages [79] and Full Information Maximum Likelihood estimation for structural equation modeling. In the case of non-ignorable missingness (missingness related to the outcomes under study), pattern mixture modeling [80] a method for identifying and incorporating patterns of missingness due to non-random drop out, will be used.

Mediation hypotheses (sub-aim 2a) will be tested within a structural equation modeling framework in a series of latent growth longitudinal mediation models [81]. The strength of longitudinal mediation approaches is the ability to use prior time points to predict future time points, thereby eliminating many of the interpretational problems in determining causal direction that occur when testing mediation with cross-sectional data [82]. Similar to the mixed-effects models described above, intercepts and slopes across time will be estimated for all longitudinal variables involved in a proposed mediation (e.g., intervention group → social support → physical activity). This model can then be specified to test causal relationships forward in time. Additional models with different, or even the same, time points at each stage can be fit to determine the strength and timing of any mediation effects. Point and interval estimates (95% CIs) of the mediating (indirect) effects of these paths for each outcome and mediator will be estimated. These models will also take into account higher levels of nesting (e.g., towns) and include important covariates as appropriate.

#### Aim 3

Evaluate program implementation and cost-effectiveness

The implementation evaluation is based upon the Consolidated Framework for Implementation Research (CFIR) and the Socioecological Model (SEM). The CFIR, derived through a comprehensive synthesis of the implementation literature [83], has five domains: inner setting, outer setting, characteristics of individuals involved, implementation processes, and intervention characteristics. Together, CFIR and the SEM will guide the assessment and help identify factors influencing implementation and intervention effectiveness. Table 4 outlines the CFIR domains and data sources. We expect a complex set of factors will distinguish high-versus-low change in study process and outcome measures. We will identify what influences the extent to which a library is able to implement SIU or Combined program and participants are able to make and maintain behavior changes. We will identify setting/organization (e.g., organizational culture, leadership) and participant (e.g., resources, priorities) factors that distinguish high/low-change among participants and why. We will identify key organizational and local factors that contribute to sustainability and scale-up [84].

**Table 4:** Implementation evaluation

CFIR Domains & Constructs	Measures/Data Sources (Timing)
***Program Characteristics*** Complexity; design; adaptability; sustainability; cost; relative advantage	• Cost analysis • Surveys: librarians (3, 6, 12 months) • Interviews: librarians (6 months) • Observations: walks/sessions, webinars
***Inner Setting*** Climate, relative priority, compatibility, leadership	• Surveys: librarians (3, 6, 12 months) • Interviews: librarians (6 months) • Observations: walks/sessions, webinars
***Outer Setting*** Participant resources, needs, facilitators & barriers; cosmopolitanism; built environment/policy	• Surveys: librarians (3, 6, 12 months), participants (baseline, 6, 12, 24 months) • Interviews: librarians (6 months), participants (high & low changers, 6, 12, 24 months) • Attendance records • Observations: walks/sessions, webinars
***Individual Characteristics*** Knowledge; attitudes & beliefs; personal attributes	• Surveys: librarians (3, 6, 12 months) • Interviews: librarians (6 months) • Observations: walks/sessions, webinars
***Process*** Planning; engaging; executing; reflecting; evaluating	• Surveys: librarians (3, 6, 12 months) • Interviews: librarians (6 months) • Observations: walks/sessions, webinars • Recruitment & retention tracking

CFIR: Consolidated Framework for Implementation Research

We will select a sample of participants in three waves for audio-recorded 40-60-minute semi-structured interviews. In wave 1 we will focus on adoption. After six months, we will identify 10 participants with positive deviance (greater increase in physical activity and improvement in fitness), high changers, and compare them with 10 participants who have lower changes in these outcomes, low changers. Participants will be selected to maximize variation in location, arm, and attendance to ensure greatest learning. In wave 2, we will focus on understanding sustainability. We will select participants with positive deviance at six months who sustained changes at 12 months, and participants with positive deviance at six months but who did not sustain changes. In wave 3, we will select participants who have sustained change for 24 months. Interviews will explore participants’ experiences with the program, asking open-ended questions about participant, program, and contextual factors that influenced engagement, behavior change, and sustainability of changes. Interviews will explore barriers and facilitators for engaging in the program, social influences (e.g., support, cohesion), environmental influences (e.g., perceptions of community, built environment), what was most useful and why, what was liked least and most, and what behavior changes were made and why. For civic engagement, we will also ask about approaches to the identified project.

### Data analysis

For quantitative data we will calculate standard descriptive statistics of frequency, percentages, central tendency, and dispersion.

Qualitative analysis will follow an immersion-crystallization approach [85], using Miller and Crabtree’s five-phase strategy [86]. This process includes (1) Describe: initial reading of the data (immersion) to identify patterns for organizing (crystallization) and summarizing of factors that shape program delivery and participant experiences; (2) Organize: coding data, creating a coding structure and organizing by themes; (3) Connect: comparing across cases, identifying factors that discriminate cases, summarizing findings with survey data to describe variations by setting/season/group, and creating a matrix [87] summarizing presence/absence factors that influence behavior change; (4) Corroborate/legitimatize: confirming/disconfirming findings with other data sources, and (5) Represent the account: identifying meaningful ways to share findings with target audiences. Initial classifications will be shared with leaders and selected participants for “member checking,” a qualitative research verification accomplished by asking key informants to confirm findings are reasonable [88–91].

### Cost effectiveness analysis

Cost-effectiveness analysis (CEA) will be conducted to evaluate and compare the costs and health gains among different arms. The CEA will utilize the incremental cost-effectiveness ratios (ICERs), defined as the incremental costs of implementing the intervention program over the improvement in the health outcomes in the community, as the primary measure for comparing the interventions. For the incremental costs, we will collect costs directly related to the program intervention excluding research-specific costs. The costs of each component, including recruitment, intervention, retention, and maintenance, of the program will be calculated separately. Improvement in health outcomes will be measured in both natural units (such as increase in physical activity and cardiovascular fitness) as well as quality-adjusted life year (QALY). We will conduct probabilistic sensitivity analysis to characterize parameter uncertainty in the calculation of ICERs and QALYs. Monte Carlo simulations based on those probability distributions will be used to obtain a joint distribution of costs and impacts in different scenarios in order to calculate ICERs and QALYs. We will use the simulated results to generate a cost-effectiveness acceptability curve to compare the probability that an intervention is acceptable for different range of willingness-to-pay thresholds and identify which intervention program is best in this particular rural population setting. CEAs will be conducted from program, healthcare sector, and societal perspectives. We will measure costs of resources used in intervention administration and implementation. Cost categories include facilities (meeting space), wages, supplies, travel, and training. For the societal perspective, we will measure direct costs included in the payer-perspective cost analysis, plus the opportunity costs, including those that impact participants.

## Discussion

Although rural residents face health disparities compared to urban residents in terms of chronic disease, there is a major gap in achieving reach and sustainable impact in behavior changes to reduce the risk of these diseases. Multilevel physical activity interventions are likely to be more effective, but there is limited evidence on how they work with rural communities, which often lack the capacity to conduct and sustain these interventions. Thus, disparities in rural physical activity persist. In this regard, the proposed study will (1) create synergy by combining two interventions to address individual, social, and community factors of physical activity; (2) conduct a mediation analysis to understand underlying mechanisms for behavior change; (3) build capacity of rural libraries for implementation and assessing this sustainable model of implementation, and (4) conduct a positive deviance analysis to understand experiences of high and low changers on key outcomes and using comparative analysis to identify factors that influence outcomes. These approaches will provide insight into how multilevel physical activity interventions work with rural residents and how they can be sustained in rural communities.

This study has several strengths. It is building the capacity of rural libraries to implement an evidence-based walking program as well as other health promotion programs in their rural communities. We will build evidence regarding the cost effectiveness of multilevel physical activity interventions targeting rural communities. We will learn what works and how these multilevel interventions work with rural populations, which experience health inequities.

There are potential limitations to this study. Recruitment and retention of rural populations can be challenging; however, we will use best practices that have been successful with rural populations [92–94]. There could be turnover of the librarians, especially in small rural libraries with limited budgets. In that event, we would provide the trainings to new librarians and provide one-to-one assistance to support the librarian in getting up to speed with the project. It is possible that the high or low changers will be clustered within a few of the 20 groups, suggesting that the group leader and/or group dynamics are having a greater impact than other factors on the degree of individual change. In that case, we would identify high and low changers within each of the groups and interview a selection across the 20 groups in order to have representation across groups. This would also allow us to gain a deeper understanding of how group dynamics and leader characteristics influence change.

### Electronic supplementary material

Below is the link to the electronic supplementary material.

Supplementary Material 1

## Data Availability

Not applicable.

## List of abbreviations

CEA: cost-effectiveness analysis
CFIR: Consolidated Framework for Implementation Research
ICER: incremental cost-effectiveness ratios
ITT: intention-to-treat (ITT)
MAR: missing at random
MCAR: missing completely at random
MDE: minimum detectable effect (MDE)
PAR-Q: Physical Activity Readiness-Questionnaire
QALY: quality-adjusted life year
SCT: Social Cognitive Theory
SEM: Socioecological Model
SIU: Step It Up!

## References

[1] United States Department of Health and Human Services (2008). Physical activity guidelines for Americans.

[2] Lee IM, Shiroma EJ, Lobelo F, Puska P, Blair SN, Katzmarzyk PT (2012). Effect of physical inactivity on major non-communicable diseases worldwide: an analysis of burden of disease and life expectancy. Lancet.

[3] Whitfield GP, Carlson SA, Ussery EN, Fulton JE, Galuska DA, Petersen R (2019). Trends in meeting physical activity guidelines among urban and rural dwelling adults - United States, 2008–2017. MMWR-Morb and Mortal Wkly Rep.

[4] Abildso CG, Daily SM, Umstattd Meyer MR, Perry CK, Eyler A (2023). Prevalence of Meeting Aerobic, Muscle-Strengthening, and combined physical activity guidelines during leisure time among adults, by rural-urban classification and region - United States, 2020. MMWR Morb Mortal Wkly Rep.

[5] Befort CA, Nazir N, Perri MG. Prevalence of obesity among adults from rural and urban areas of the United States: findings from NHANES (2005–2008). J Rural Health. 2012:392–7. 10.1111/j.1748-0361.2012.00411.x PMC3481194 23083085

[6] Trivedi T, Liu J, Probst J, Merchant A, Jones S, Martin AB. Obesity and obesity-related behaviors among rural and urban adults in the USA. Rural Remote Health. 2015;15(4). 26458564

[7] Henley SJ, Invasive Cancer I. 2004–2013, and deaths, 2006–2015, in Nonmetropolitan and Metropolitan Counties—United States. MMWR Surveillance Summaries. 2017;66. 10.15585/mmwr.ss6614a1 PMC5879727 28683054

[8] Berrigan D, Carroll D, Fulton J, Galuska D, Brown D, Dorn J (2012). Vital Signs: walking among adults-United States, 2005 and 2010. MMWR Morb Mortal Wkly Rep.

[9] Ussery EN, Carlson SA, Whitfield GP, Watson KB, Berrigan D, Fulton JE (2018). Transportation and leisure walking among U.S. adults: Trends in reported prevalence and volume, National Health interview Survey 2005–2015. Am J Prev Med.

[10] Watson KB, Frederick GM, Harris CD, Carlson SA, Fulton JE (2015). U.S. adults’ participation in specific activities: behavioral risk factor surveillance System–2011. J Phys Act Health.

[11] Whitfield GP, Carlson SA, Ussery EN, Watson KB, Berrigan D, Fulton JE (2019). National-level environmental perceptions and walking among urban and rural residents: informing surveillance of walkability. Prev Med.

[12] Sallis J, Owen N. Ecoglogical models of health behavior. In: Galnz K, Rimer B, Viswanath K, editors. Health Behavior Theory, Reserach and Practice, 5th edition. San Francisco, CA: Jossey-Bass; 2015.

[13] U.S. Department of Health and Human Services (2015). Step it up! The Surgeon General’s call to action to promote walking and Walkable Communities.

[14] Centers for Disease Control and Prevention (2011). Strategies to prevent obesity and other Chronic Diseases: the CDC Guide to strategies to increase physical activity in the community.

[15] Hanson S, Jones A (2015). Is there evidence that walking groups have health benefits? A systematic review and meta-analysis. Br J Sports Med.

[16] US Preventive Services Task Force. The guide to community preventive servies. The Community Guide; 2019.

[17] Kassavou A, Turner A, French DP (2013). Do interventions to promote walking in groups increase physical activity? A meta-analysis. Int J Behav Nutr Phys Act.

[18] Perry CK, Rosenfeld AG, Bennett JA, Potempa K (2007). Heart-to-Heart: promoting walking in rural women through motivational interviewing and group support. J Cardiovasc Nurs.

[19] Pereira MA, Kriska AM, Day RD, Cauley JA, LaPorte RE, Kuller LH (1998). A randomized walking trial in postmenopausal women: effects on physical activity and health 10 years later. Arch Intern Med.

[20] Harris T, Kerry SM, Limb ES, Furness C, Wahlich C, Victor CR et al. Physical activity levels in adults and older adults 3–4 years after pedometer-based walking interventions: long-term follow-up of participants from two randomised controlled trials in UK primary care. PLoS Med. 2018;15(3). 10.1371/journal.pmed.1002526 PMC5844512 29522529

[21] Grant G, Machaczek K, Pollard N, Allmark P (2017). Walking, sustainability and health: findings from a study of a walking for Health group. Health Soc Care Community.

[22] Izumi BT, Schulz AJ, Mentz G, Israel BA, Sand SL, Reyes AG (2015). Leader Behaviors, Group Cohesion, and participation in a walking Group Program. Am J Prev Med.

[23] Seguin R, Connor L, Nelson M, LaCroix A, Eldridge G (2014). Understanding barriers and facilitators to healthy eating and active living in rural communities. J Nutr Metabolism.

[24] Wilcox S, Castro C, King AC, Housemann R, Brownson RC (2000). Determinants of leisure time physical activity in rural compared with urban older and ethnically diverse women in the United States. J Epidemiol Commun Health.

[25] Parks S, Housemann RA, Brownson RC (2003). Differential correlates of physical activity in urban and rural adults of various socioeconomic backgrounds in the United States. J Epidemiol Community Health.

[26] South J, Giuntoli G, Kinsella K, Carless D, Long J, McKenna J (2017). Walking, connecting and befriending: a qualitative pilot study of participation in a lay-led walking group intervention. J Transp Health.

[27] Lindsay-Smith G (2019). Active ageing in the community. Exploring the role of Community Activity Groups for older adults for physical activity.

[28] Durand CP, Andalib M, Dunton GF, Wolch J, Pentz MA (2011). A systematic review of built environment factors related to physical activity and obesity risk: implications for smart growth urban planning. Obes Rev.

[29] Saelens BE, Handy SL (2008). Built environment correlates of walking: a review. Med Sci Sports Exerc.

[30] Carrapatoso S, Silva P, Purakom A, Novais C, Colaço P, Carvalho J (2017). The experience of older adults in a walking program at individual, interpersonal, and environmental levels. Activities Adaptation & Aging.

[31] Schulz AJ, Israel B, Sand SL, Gamboa C, Gaines C, Reyes AG, et al. editors. Building the healthiest nation by promoting health in vulnerable communities: impact and outcome evaluation of the Walk Your Heart to Health community-based multilevel intervention. APHA 2016 Annual Meeting & Expo (Oct 29-Nov 2, 2016); 2016: American Public Health Association.

[32] Zenk SN, Wilbur J, Wang E, McDevitt J, Oh A, Block R (2009). Neighborhood environment and adherence to a walking intervention in african american women. Health Educ Behav.

[33] Adler RP, Goggin J (2005). What do we mean by civic engagement?. J Transformative Educ.

[34] Brown AGM, Hudson LB, Chui K, Metayer N, Lebron-Torres N, Seguin RA et al. Improving heart health among Black/African american women using civic engagement: a pilot study. BMC Public Health. 2017;17. 10.1186/s12889-016-3964-2 PMC5259944 28118823

[35] Seguin RA, Folta SC, Sehlke M, Nelson ME, Heidkamp-Young E, Fenton M et al. The StrongWomen Change Clubs: engaging residents to catalyze positive change in food and physical activity environments. Journal of Environmental and Public Health. 2014;2014. 10.1155/2014/162403 PMC4265724 25525441

[36] Gavin VR, Seeholzer EL, Leon JB, Chappelle SB, Sehgal AR (2015). If we build it, we will come: a model for community-led change to transform neighborhood conditions to support healthy eating and active living. Am J Public Health.

[37] Glanz K, Rimer BK, Viswanath K. Health Behavior and Health Education: theory, Research, and practice. John Wiley & Sons; 2008.

[38] Flaherty M, Miller D (2016). Rural public libraries as community change agents: opportunities for health promotion. J Educ Libr Inform Sci.

[39] Lenstra N. Let’s move! Fitness programming in public libraries. Public Libr Q.37(1):61–80.

[40] Lenstra N (2017). Movement-based programs in U.S. and canadian public libraries: evidence of impacts from an exploratory survey. Evid Based Libr Inform Pract.

[41] Lenstra N (2018). The role of public librarians in supporting physical activity: challenging the Jacks of all Trades but Masters of none librarian syndrome. Adv Libr Adm Organ.

[42] Lenstra N, Carlos J. Public libraries and walkable neighborhoods. Int J Environ Res Public Health. 2019;16(10). 10.3390/ijerph16101780 PMC6572033 31137540

[43] Philbin MM, Parker CM, Flaherty MG, Hirsch JS (2019). Public libraries: A Community-Level Resource to Advance Population Health. J Community Health.

[44] Hummel K (2022). Libraries transforming Communities. Natl Civic Rev.

[45] Brownson RC, Fielding JE, Green LW (2018). Building Capacity for evidence-based Public Health: reconciling the Pulls of Practice and the push of Research. Annu Rev Public Health.

[46] Creswell J, Plano Clark V. Designing and Conducting Mixed Methods Research, 2nd edition,. Thousand Oaks, California: Sage Publications; 2011.

[47] Pelczar M, Soffronoff J, Nielsen E, Li J, Mabile S (2022). Data file documentation: public libraries in the United States Fiscal Year 2020.

[48] Sturdevant SG, Huang SS, Platt R, Kleinman K (2021). Matching in cluster randomized trials using the Goldilocks Approach. Contemp Clin Trials Commun.

[49] Public Health Foundation. Competency Assessment. Tier 1 Public Health Professionals. Council on Linkages between Academia and Public Health Practice; 2014.

[50] Thomas S, Reading J, Shephard R (1992). Revision of the physical activity readiness questionnaire (PAR-Q). Candian J Sports Sci.

[51] Bandura A (1997). Self-efficacy the exercise of control.

[52] Carron A, Hausenblas HA, Eastbrooks P, Bull S (1999). Social influence and exercise involvement. Adherence issues in sport and exercise.

[53] Ryan RM, Deci EL (2000). Self-determination theory and the facilitation of intrinsic motivation, social development, and well-being. Am Psychol.

[54] Carron A, Hausenblas HA (1998). Group Dynamics in Sport.

[55] Baranowski T, Perry C, Parcel GS, Glanz K, Lewis FM, Rimer B (1997). How individuals, environments, and health behavior interact. Health Behavior and Health Education Theory, Research and Practice.

[56] Deci EL, Ryan RM (1985). Intrinsic motivation and self-determination in human behavior.

[57] Williams SL, French DP (2011). What are the most effective intervention techniques for changing physical activity self-efficacy and physical activity behaviour–and are they the same?. Health Educ Res.

[58] Carron AV, Hausenblas HA, Mack D (1996). Social influence and exercise: a meta-analysis. J Sport Exerc Psychol.

[59] Pavey TG, Gilson ND, Gomersall SR, Clark B, Trost SG (2017). Field evaluation of a random forest activity classifier for wrist-worn accelerometer data. J Sci Med Sport.

[60] Ahmadi MN, Nathan N, Sutherland R, Wolfenden L, Trost SG (2020). Non-wear or sleep? Evaluation of five non-wear detection algorithms for raw accelerometer data. J Sports Sci.

[61] Trost S, Mciver K, Pate R (2005). Conducting accelerometer-based activity assessments in field-based research. Med Sci Sports Exerc.

[62] Crapo RO, Casaburi R, Coates AL, Enright PL, MacIntyre NR, McKay RT (2002). ATS statement: guidelines for the six-minute walk test. Am J Respir Crit Care Med.

[63] Sampson RJ, Raudenbush SW, Earls F (1997). Neighborhoods and violent crime: a multilevel study of collective efficacy. Science.

[64] Brazier J, Roberts J, Deverill M (2002). The estimation of a preference-based measure of health from the SF-36. J Health Econ.

[65] Centers for Disease Control and Prevention. Behaviroal Rsk Factro Surveillance System 2021 [Available from: https://www.cdc.gov/brfss/about/about_brfss.htm.

[66] Craig CL, Marshall AL, Sjostrom M, Bauman AE, Booth ML, Ainsworth BE (2003). International physical activity questionnaire: 12-country reliability and validity. Med Sci Sports Exerc.

[67] Marcus BH, Selby VC, Niaura RS, Rossi JS (1992). Self-efficacy and the stages of exercise behavior change. Res Q Exerc Sport.

[68] Estabrooks P, The Physical Activity Group Environment Questionnaire (2000). An instrument for the assessment of cohesion in exercise classes. Group Dynamics: Theory Research and Practice.

[69] Sallis JF, Grossman RM, Pinski RB, Patterson TL, Nader PR (1987). The development of scales to measure social support for diet and exercise behaviors. Prev Med.

[70] Jakes S, Shannon L. Mobilization Scale - Individual 2002 [Available from: https://cals.arizona.edu/sfcs/cyfernet/nowg/MobilizationsScaleIndividualPacket.pdf.

[71] Doolittle A, Faul A (2013). Civic engagement scale: a validation study. Sage Open.

[72] Ball K, Jeffery RW, Abbott G, McNaughton SA, Crawford D (2010). Is healthy behavior contagious: associations of social norms with physical activity and healthy eating. Int J Behav Nutr Phys Act.

[73] Mujahid MS, Diez Roux AV, Morenoff JD, Raghunathan T (2007). Assessing the measurement properties of neighborhood scales: from psychometrics to ecometrics. Am J Epidemiol.

[74] Natural England. The People and Nature Survey 2020 [Available from: https://www.gov.uk/government/collections/people-and-nature-survey-for-england.

[75] Raudenbush SW et al. Optimal Design Software for Multi-level and Longitudinal Research (Version 3.01). 2011.

[76] Harris PA, Taylor R, Minor BL, Elliott V, Fernandez M, O’Neal L (2019). The REDCap consortium: building an international community of software platform partners. J Biomed Inform.

[77] Benjamini Y, Hochberg Y (1995). Controlling the false discovery rate: a practical and powerful approach to multiple testing. J Royal Stat Soc Ser B.

[78] Little RJA, Rubin DB (2002). Statistical analysis with Missing Data Second Edition.

[79] Kenward M, Carpenter J (2007). Multiple imputation: current perspectives. Stat Methods Med Res.

[80] Hedeker D, Gibbons R (1997). Application of random-effects pattern-mixture models for missing data in longitudinal studies. Psychol Methods.

[81] Selig JP, Preacher K (2009). Mediation models for longitudinal data in developmental research. Res Hum Dev.

[82] Gollob H, Reichardt C (1987). Taking account of time lags in causal models. Child Dev.

[83] Damschroder LJ, Aron DC, Keith RE, Kirsh SR, Alexander JA, Lowery JC (2009). Fostering implementation of health services research findings into practice: a consolidated framework for advancing implementation science. Implement Sci.

[84] World Health Organization (2016). Scaling up projects and initiatives for better health: from concepts to practice.

[85] Borkan J, Crabtree BF, Miller WL (1999). Immersion/crystallization. Doing qualitative research.

[86] Cunningham BA, Bonham VL, Sellers SL, Yeh HC, Cooper LA (2014). Physicians’ anxiety due to uncertainty and use of race in medical decision making. Med Care.

[87] Miles MB, Huberman AM (1994). Qualitative data analysis: an expanded sourcebook.

[88] Ghanbarzadeh R, Ghapanchi AH, Blumenstein M, Talaei-Khoei A (2014). A decade of research on the use of three-dimensional virtual worlds in health care: a systematic literature review. J Med Internet Res.

[89] Lincoln Y, Guba EG, Guba EG (1985). Naturalistic inquiry.

[90] Nagendran M, Gurusamy KS, Aggarwal R, Loizidou M, Davidson BR (2013). Virtual reality training for surgical trainees in laparoscopic surgery. Cochrane Database Syst Rev.

[91] Hoffart N (1991). A member check procedure to enhance rigor in naturalistic research. West J Nurs Res.

[92] Martinez CR, McClure HH, Eddy JM, Ruth B, Hyers MJ (2012). Recruitment and retention of latino immigrant families in prevention research. Prev Sci.

[93] Reidy MC, Orpinas P, Davis M (2012). Successful recruitment and retention of latino study participants. Health Promot Pract.

[94] Young L, Barnason S, Do V. Review strategies to Recruit and Retain Rural patient participating self-management Behav Trials. 2015;10(2). 10.4148/1936-0487.1070 PMC5451124 28580049

